# Effective population management practices in diabetes care - an observational study

**DOI:** 10.1186/1472-6963-10-277

**Published:** 2010-09-21

**Authors:** Anne Frølich, Jim Bellows, Bo Friis Nielsen, Per Bruun Brockhoff, Martin Hefford

**Affiliations:** 1 Department of Integrated Healthcare, Bispebjerg Hospital, Copenhagen, Denmark; 2 Care Management Institute, Kaiser Permanente, Oakland, California, USA; 3 DTU Informatics, Technical University of Denmark, Kgs. Lyngby, Denmark; 4 Hutt Valley District Health Board, Lower Hutt, New Zealand

## Abstract

**Background:**

Ensuring that evidence based medicine reaches patients with diabetes in the US and internationally is challenging. The chronic care model includes evidence based management practices which support evidence based care. However, despite numerous studies, it is unclear which practices are most effective. Few studies assess the effect of simultaneous practices implemented to varying degrees. The present study evaluates the effect of fifteen practices applied concurrently and takes variation in implementation levels into account while assessing the impact of diabetes care management practices on glycemic and lipid monitoring.

**Methods:**

Fifteen management practices were identified. Implementation levels of the practices in 41 medical centres caring for 553,556 adults with diabetes were assessed from structured interviews with key informants. Stepwise logistic regression models with management practices as explanatory variables and glycemic and lipid monitoring as outcome variables were used to identify the diabetes care practices most associated with high performance.

**Results:**

Of the 15 practices studied, only provider alerts were significantly associated with higher glycemic and lipid monitoring rates. The odds ratio for glycemic monitoring was 4.07 (p < 0.00001); the odds ratio for lipid monitoring was 1.63 (p < 0.006). Weaker associations were found between action plans and glycemic monitoring (odds ratio = 1.44; p < 0.03) and between guideline distribution and training and lipid monitoring (odds ratio = 1.46; p < 0.03). The covariates of gender, age, cardiac disease and depression significantly affected monitoring rates.

**Conclusions:**

Of fifteen diabetes care management practices, our data indicate that high performance is most associated with provider alerts and more weakly associated with action plans and with guideline distribution and training. Lack of convergence in the literature on effective care management practices suggests that factors contributing to high performance may be highly context-dependent or that the factors involved may be too numerous or their implementation too nuanced to be reliably identified in observational studies.

## Background

Diabetes mellitus is among the leading chronic diseases in the US and internationally [1,2]. Complications of diabetes can be reduced through appropriate medical care and behavior modification [3,4]. Ensuring that patients with diabetes receive evidence based care to control disease and reduce the risk of complications is a significant challenge; much evidence exists of the gap between optimal care and the actual care patients receive [5,6]. The chronic care model advocates an evidence based approach for disease management [7-9]. The model proposes several evidence based management practices organised in six interrelated elements supporting the implementation of evidence based medicine [10,11].

It is essential to know which combinations of practices are most effective at improving diabetes care. Results from meta-analyses, randomized controlled trials, reviews and observational studies evaluating the impact of management practices on diabetes care are conflicting, presenting divergent results; important issues regarding the chronic care model and the practices embedded within it remain unresolved [12-16].

A systematic review assessing the effect of management practices in diabetes care concluded that professional and organizational practices, individually and in combination, improved diabetes care processes [17]. Adding patient education and or improving the role of a nurse led to improved care outcomes. Few studies assess the effects of individual management practices when several practices are applied at the same time and analysed with multivariate statistical models [14-16,18-21].

Kerr has recommended that conflicting results among observational studies of care management could be addressed via a study design in which specific practices are carefully defined and the degree of practice implementation is consistently measured across diverse settings and compared to the outcomes delivered. The study reported here implements that approach [22].

We studied care management practices in a large U.S. integrated health care delivery system that has been recognized as a relatively high quality provider. It provides comprehensive care to several million members, including more than 500,000 adults with diabetes. Within this organisation, decentralized operating units result in variability in the way components of the chronic care model are implemented and in the relative emphasis on each component. Implementation varies between centers and for individual components within centers; consequently, local population care programs differ across the system.

The organisation has invested in standardized measurement of numerous quality indicators to enable comparisons over time and across operating units. Internal reporting shows variations in performance on process and outcome measures. Variability in population care practices and performance, together with standardized measurements, provide a ripe opportunity for learning about the impact of the chronic care model on diabetes care processes.

The purpose of this study was to identify management practices that affect glycemic and lipid monitoring in diabetes care in the context of several concurrent care management practices implemented at varying levels. We chose to use monitoring rates as our outcomes as they are solely a function of provider decision-making.

## Methods

We conducted a cross-sectional observational study. We identified fifteen diabetes population care management practices by reviewing the literature on diabetes care and the Chronic Care Model. These are briefly described in Table 1. The extent of implementation of each practice was assessed at each medical center by a survey administered to a key informant. Survey items were mainly drawn or adapted from existing instruments, including the Assessment of Chronic Illness Care, the National Survey of Physician Organizations, and the Translating Research into Action for Diabetes instruments [23-25].

**Table 1:** Population care practices surveyed.

Practice	Definition	Items
Health System Organization		
Financial Incentives	Amount of physician salary at risk, subject to assessment of performance on population care quality indicators.	4
Provider Feedback	Reports to providers about performance and degree of blinding to all providers.	40
Self-Management Support		
Patient Action Plans	Individual goal setting supported by action plans including needs assessment, personalization, and regular clinician review.	4
Patient Education	Education and support services based on self-management principles in a variety of formats.	35
Delivery System Design		
Defined Care Path	An explicit protocol or model guides population care.	4
Risk Stratification	Use of an algorithm to stratify patients by risk level and determine the level of proactive care provided.	4
Outreach/Follow-Up	Proactive, planned care.	19
Inreach	Customized reminders for patients of needed care whenever they present for service.	5
Care Coordination	Processes and structures supporting effective patient care handoffs, including explicit protocols and accountabilities.	6
Cultural Competence	Care tailored to the needs of major racial, ethnic, and cultural groups.	15
Team Accountability	Accountability for patient care vested in care teams rather than individuals.	1
Decision Support		
Guideline Distribution and Training	Distribution of evidence-based guidelines and clinician training on guideline content, including electronic availability, continuing medical education, and inter-provider communications.	5
Provider Alerts	Customized, context-sensitive paper-based or electronic alerts reminding providers of appropriate care for individual patients and groups of patients.	28
Clinical Information Systems		
Registry	Completeness and quality of a registry or database of key indicators for all patients with diabetes.	72
Electronic Medical Record	Availability and comprehensiveness of clinical data during patient visits.	36

We interviewed one key informant at each of the 41 sites at the integrated health care delivery system. Most key informants were non-physician managers responsible for population based care or diabetes care; some were physician champions for diabetes care. Survey items elicited factual information rather than beliefs or opinions, and key informants were well positioned to provide the requested information.

We developed algorithms to summarize detailed survey information into fifteen summary scores representing distinct population management practices. Three population care experts blinded to the data weighted individual items to form summary practice scores ranging from a minimum of 0 to a maximum of 1. More extensive implementation resulted in a higher score. The range of implementation scores for 15 population management practices across 41 sites is shown in Table 2. Data was registered one year after the interviews took place, as we wanted to be sure that the practices were well-implemented and had exerted their effects.

**Table 2:** Variation in diabetes population care practice scores across sites.

Care Management Practices	Mean	Low Score	High Score	Standard Deviation
Health System Organization				
Financial Incentives	0.01	0.00	0.60	0.07
Provider Feedback	0.53	0.00	1.00	0.30
Self-Management Support				
Patient Action Plans	0.37	0.00	0.77	0.19
Patient Education	0.61	0.17	0.91	0.17
Delivery System Design				
Defined Care Path	0.37	0.08	0.70	0.14
Risk Stratification	0.82	0.17	1.00	0.21
Outreach and Follow-Up	0.74	0.38	0.84	0.14
Inreach	0.57	0.00	1.00	0.43
Care Coordination	0.70	0.10	1.00	0.26
Cultural Competence	0.72	0.20	1.00	0.22
Team Accountability	0.63	0.00	1.00	0.34
Decision Support				
Guideline Distribution and Training	0.73	0.23	1.00	0.18
Provider Alerts	0.58	0.00	1.00	0.22
Clinical Information Systems				
Registry	0.71	0.64	0.81	0.06
Electronic Medical Record	0.70	0.00	1.00	0.46

Note: Average performance cannot be meaningfully compared between practices due to practice-specific scoring algorithms

Glycemic monitoring rate was defined as the percentage of members with diabetes who had one or more glycated hemoglobin (HbA1c) tests during one calendar year. Lipid monitoring rate was defined as the percentage of members with diabetes who had one or more low density lipoprotein-C (LDL-C) tests during one calendar year.

Person-level data on monitoring status were aggregated into strata defined by medical center (41 categories), age group (4 categories), gender, presence/absence of depression, and presence/absence of cardiovascular disease, and monitoring rates were calculated for all strata. Of the 1312 possible location-age-gender-comorbidity combinations, 1198 combinations were populated by one or more individuals. These 1198 strata were the units of observation for our analyses, with the outcome variables expressed as monitoring rates.

As we had only 41 medical centres and a large number of explanatory variables, we used stepwise regression analysis to obtain reasonably parsimonious models that were not overly parameterized. Care management practices were used as explanatory variables in a forward selection, stepwise logistic regression model, with random effects of medical centers and the observation level and dependent variables of glycemic and lipid monitoring rates. The medical center random effect captured excess variability between the 41 medical centers and played a major role in the statistical analysis in that it accounted for the medical center clustering effect of observations, hence providing the proper unbiased error estimates. The medical center random effect captured excess variability between the 41 medical centers and played a major role in the statistical analysis. The observation level random effect captured overdispersion in the data stemming from the fact that the effects of the independent variables and covariates do not completely explain the excess variation in outcomes [26]. The random effects associated with medical centres and observation levels were similar in both models, with rather high and comparable dispersion.

Each outcome was modeled separately, assessing the degree of association between various practices and the specific process measure. The models were fitted and compared by maximum likelihood methods using the "lmer" function of R. Even though we had a large number of explanatory variables and relatively few medical centers, our analysis did not demonstrate multicollinearity to an extent that would have made our models unstable.

## Results

The 41 practice sites provided care for 553,556 adults with diabetes, 51% of which were male. Among patients with diabetes, 16% were also diagnosed with coronary artery disease (CAD) and 13% with depression. Four percent of patients with diabetes were 18 to 34 years of age, 18% were 35 to 49 years old, 38% were 50 to 64 years old, and 40% were aged 65 years and up. The mean HbA1c was 7.2% (55 mmol/mol) and the mean LDL-C level was 105.1 mg/dl (2.72 mmol/L).

The regression models showed that provider alerts significantly affected the likelihood of both glycemic and lipid monitoring. This care management practice, in which providers received reminders of appropriate care delivered as computerized prompts or paper chart attachments, had a strong effect on both glycemic and lipid monitoring rates, increasing the odds ratios for glycemic monitoring by 4.07 (p < 0.00001) and for lipid monitoring by 1.63 (p < 0.0006) (Table 3). Sites that scored highest on this practice had automated, computerized alerts integrated into electronic medical records.

**Table 3:** Parameter estimates, odds ratios, and P-values for the three significant management practices.

	Glycemic monitoring model			Lipid monitoring model		
	Parameter estimate	Odds ratio	P-value	Parameter estimate	Odds ratio	P-value
**Provider alert**	1,4	4,07	> 0.00001	0,49	1,63	0.0006
**Guideline distribution and training**	0,38	1,46	0.03			
**Action plans**				0,36	1,44	0.03

Practices not significantly related to monitoring outcomes are omitted.

Two other practices affected monitoring rates. Guideline distribution and training increased the likelihood of glycemic monitoring; the odds ratio was 1.46 (p < 0.03). Action plans increased the likelihood of lipid monitoring; the odds ratio was 1.44 (p < 0.03).

The covariates of gender, age, CAD, and depression affected monitoring rates in both models. Gender differences for monitoring rates decreased with increasing age (Table 4). The combined effect of depression and age resulted in statistically higher odds ratios for both types of monitoring for members up to 64 years of age, while odds ratios were lower for those who were 65 or older (0.95 and 0.87) (Table 5). The combined effect of CAD and age only affected the likelihood of glycemic monitoring in adults between the ages of 50 and 64, the odds ratio was 1.18 (p < 0.0001). The combined effect of CAD and gender increased the likelihood of lipid monitoring significantly in both men and women.

**Table 4:** Parameter estimates, odds ratios, and P-values for age and sex and their interactions.

	Glycemic monitoring model			Lipid monitoring model		
	Parameter estimate	Odds ratio	P-value	Parameter estimate	Odds ratio	P-value
**Age 18-34**	-0,63	0,53	0.0003	-0,07	0,93	0.52
**Age 35-49**	-0,23	0,8	0.18	0,53	1,7	< 0.0001
**Age 50-64**	0,12	1,13	0.49	0,98	2,67	< 0.0001
**Age 65up**	0,29	1,34	0.09	1,2	3,32	< 0.0001
						
**Age 18-34 female**	-0,56	0,57	< 0.0001	-0,47	0,62	< 0.0001
**Age 35-49 female**	-0,17	0,84	< 0.0001	-0,13	0,88	< 0.0001
**Age 50-64 female**	0,08	1,08	0.005	0,09	1,1	0.0006
**Age 65up female**	-0,08	0,93	0.002	-0,06	0,94	0.02

**Table 5:** Parameter estimates, odds ratios and P-values for age and sex and comorbidities the three significant management practices.

	Glycemic monitoring model			Lipid monitoring model		
	Parameter estimate	Odds ratio	P-value	Parameter estimate	Odds ratio	P-value
**Age 18-34 and depression**	0,3	1,34	< 0.0001	0,29	1,33	< 0.0001
**Age 35-49 and depression**	0,34	1,41	< 0.0001	0,31	1,37	< 0.0001
**Age 50-64 and depression**	0,19	1,21	< 0.0001	0,22	1,25	< 0.0001
**Age 65up and depression**	-0,05	0,95	0.08	-0,14	0,87	< 0.0001
						
**Age 18-34 and CAD**	0,2	1,22	0.41			
**Age 35-49 and CAD**	0,11	1,11	0.03			
**Age 50-64 and CAD**	0,17	1,18	< 0.0001			
**Age 65up and CAD**	-0,02	0,98	0.39			
						
**Female and CAD**				0,74	2,1	< 0.0001
**Female and CAD**				0,51	1,67	< 0.0001

Coronary artery disease (CAD).

In summary, the models for glycemic and lipid monitoring showed highly consistent effects; we interpret this finding as genuine because the implementation level of provider alerts differed across sites for these outcomes.

## Discussion

We assessed the individual effects of fifteen concurrently and variably implemented population care practices on two diabetes process of care measures. Provider alerts increased the likelihood of glycemic and lipid monitoring to a statistically significant degree. The effects of action plans and guideline distribution and training were significant at a level well below that of provider alerts.

Other studies support our finding that female gender is associated with lower quality of care for diabetes and/or cardiovascular disease [27-29]. Notably, we found that gender differences disappeared with increasing age. In our study, increasing age was markedly associated with higher monitoring rates, contradicting evidence that it is associated with poorer quality of care [30]. Overall, our data suggest that care might be more strongly affected by patient characteristics than by guideline recommendations.

Strengths of our study included the very large population managed at the care sites we studied and our ability to quantify the effect of practices taking implementation levels into account as recommended by Kerr [22]. Limitations to our study included the relatively narrow variation across sites; homogeneity of care management practices throughout the integrated health care delivery system stems from multiple factors, including clustering of sites within operationally consistent regions.

Other limitations are inherent in the observational study design, including the difficulty of measuring implementation levels and the large number of unmeasured factors and interactions that influence outcomes in real-world settings. Measuring implementation levels is a challenging endeavour about which relatively little has been written [14,15]. More interviews with different health professionals with a variety of competencies and understandings of provided care might have improved the understanding of the implementation level of the management practices. However, because the interviews covered diverse aspects of care, many respondents gathered information from colleagues in preparation for the interview, so the single interviews typically integrate input from several individuals. We generally found that we could assess the degree to which structures and processes were in place to support each practice, but not their quality, highlighting the possibility of measurement error. To understand the practices in greater detail, we also conducted a series of case studies on diabetes care at four sites [31]. However, we were unable to complete a detailed assessment at all 41 sites.

Our results are best interpreted in the context of previous related studies. Provider alerts creating point-of-care reminders have been previously identified as an effective strategy for improving diabetes care [14,16,17,32-34]. Varying effects of action plans and guideline distribution and training are shown in the literature [14,16,17,35-38].

Another study using multivariate statistical modelling, The Translating Research into Action for Diabetes (TRIAD) study of approximately 9000 patients, examined the association between intensity of disease management by physicians and diabetes process and outcomes measures [25]. The TRIAD study identified an association between physician reminders and both lipid and glycemic monitoring rates: however, the same effect was noted for two other practices, structured care management and performance feedback, for which we did not find an association with monitoring rates.

In a multivariate study conducted in the U.S. Veterans Health Administration, four other practices affected a composite process measure of quality (monitoring tests and physical examinations): the level of support for guideline efforts, regional office leadership for guideline use, hospital use of guideline performance data, and hospital culture [16]. Other studies assessing the impact of management practices on diabetes quality of care and relying on multivariate statistical models have identified a variety of practices as important to quality [15,16,18-21]. Financial incentives have attracted much attention but did not have significant impact on the screening rates for HbA1c values nor for LDL-Cholesterol. Recent studies show mixed results of financial incentives regarding quality of care improvements [39-41].The physician financial incentives in place at the time of this study were small (less than 3% of total physician income). In sum, the literature is characterized by inconsistent findings regarding effective management practices in diabetes care.

Several factors may explain the lack of conclusive evidence. Comparability between studies is poor. Definitions of care management practices vary, and the Cochrane Collaboration suggests aligning these [32]. However, even across our study sites, we were not able to ensure consistency in the operational definitions of care management practices because they had been developed at the local level within the integrated health care delivery system over many years.

Another cause of conflicting results in the existing literature may be the effect of organisational and cultural contexts on the frontline delivery of diabetes care. These factors are very challenging to measure and analyze, and we did not attempt to do so.

Future research identifying effective management practices should build on the methods we used, including measurement of implementation levels and multivariate statistical models to assess the effects of multiple practices implemented in combination. Future efforts should also seek to align definitions of management practices, implementation levels, and outcome variables. The effects of patient-level covariates and organisational context should be taken into consideration.

The purpose of research attempting to identify specific practices most associated with better outcomes is to inform future quality improvement efforts; scarce resources would arguably be most efficiently allocated to those practices. This vein of research essentially attempts to "reverse engineer" critical performance drivers so they can be disseminated broadly or further amplified. However, conflicting results from observational care management studies suggest that this approach may not be the best way to identify or prioritize improvement opportunities; context dependencies and implementation details may preclude identifying performance drivers via observational studies. Quality improvement teams should consider other approaches. Two sound alternatives are based on "learning from improvement": using rapid-cycle improvement methods and/or identifying high-performing sites with similar organisational contexts from which structures and processes can be adopted [42].

## Conclusions

The diabetes care management practice of provider alerts improved glycemic and lipid monitoring rates in patients with diabetes. Action plans and guideline distribution and training also affected monitoring rates, although to a lesser degree. The covariates of gender, age, CAD and depression also impacted monitoring rates to a statistically significant degree, individually and in combination.

Our findings contribute to a knowledge base that contains somewhat sparse and often conflicting results. Observational studies with uncontrolled variation on multiple factors may be inadequate for identifying the practices that could contribute most to ongoing improvement in population care, and quality improvement teams should also consider other methods for identifying improvement opportunities.

## Ethical approval

The study was reviewed and approved by the Institutional Review Boards for KP Northern California, KP Southern California, KP Northwest, KP Georgia, and KP Hawaii.

## Competing interests

The authors declare that they have no competing interests.

## Authors' contributions

AF designed the concept and conduct of the study and interpreted data, drafted the manuscript, and participated in the decision to submit it. JB designed the concept and conduct of the study, collected, analyzed and interpreted data, drafted the manuscript and participated in the decision to submit it. BFN conducted the data analysis and interpreted findings and helped to draft the manuscript. PBB conducted the data analysis and interpreted findings. MH interpreted data and drafted the manuscript. AF, JB, BFN, PBB, and MH read, commented, and approved the final version of the manuscript.

## Pre-publication history

The pre-publication history for this paper can be accessed here:

http://www.biomedcentral.com/1472-6963/10/277/prepub
